# Avaliação Cintilográfica e Ecocardiográfica em Portadores de Variantes Patogênicas ou Provavelmente Patogênicas do Gene TTR sem Envolvimento Cardíaco Manifesto

**DOI:** 10.36660/abc.20230216

**Published:** 2024-05-07

**Authors:** Tonnison de Oliveira Silva, Eduardo Sahade Darzé, Marcela Machado Costa, Luiz José Junior, Antonio A. B. Ximenes, Fábio Fernandes, Mário de Seixas Rocha, Marcia M. Noya-Rabelo, Luiz Eduardo Fonteles Ritt

**Affiliations:** 1 Hospital Cardio Pulmonar Instituto D'Or de Ensino e Pesquisa Salvador BA Brasil Hospital Cardio Pulmonar - Instituto D'Or de Ensino e Pesquisa – IDOR, Salvador, BA – Brasil; 2 Escola Bahiana de Medicina e Súde Pública Salvador BA Brasil Escola Bahiana de Medicina e Súde Pública, Salvador, BA – Brasil; 3 Instituto do Coração do Hospital das Clínicas da Faculdade de Medicina da Universidade de São Paulo São Paulo SP Brasil Instituto do Coração do Hospital das Clínicas da Faculdade de Medicina da Universidade de São Paulo, São Paulo, SP – Brasil; 4 Hospital São Rafael Instituto D'Or de Ensino e Pesquisa Salvador BA Brasil Hospital São Rafael - Instituto D'Or de Ensino e Pesquisa – IDOR, Salvador, BA – Brasil

**Keywords:** Envolvimento Cardíaco Precoce, Amiloidose por Transtirretina, Cintilografia com Pirofosfato, Ecocardiografia com *Strain*

## Abstract

**Fundamento:**

A amiloidose por transtirretina (ATTR) é uma doença infiltrativa causada pela deposição anormal de proteína principalmente no coração e no sistema nervoso periférico. Quando acomete o coração, a doença manifesta-se como uma cardiomiopatia restritiva e, quando afeta o sistema nervoso periférico e autônomo, apresenta-se como uma polineuropatia, podendo ser chamada de Polineuropatia Amiloidótica Familiar (PAF). Existem dois subtipos de ATTR, a ATTR selvagem, em que não há variantes genéticas, e a ATTR hereditária, caracterizada por uma variante no gene que codifica a proteína transtirretina (T\TR). Em ambos os subtipos, o envolvimento cardíaco é o principal marcador prognóstico.

**Objetivos:**

Avaliar a prevalência do envolvimento cardíaco subclínico em uma amostra de pacientes com variantes genéticas no gene TTR usando a cintilografia com pirofosfato e o ecocardiograma com *strain* ; comparar os achados cintilográficos e as medidas de *strain* ; avaliar a associação entre PAF e o envolvimento subclínico; e analisar se existe uma associação entre uma variante genética específica e o envolvimento cardíaco.

**Métodos:**

Estudo transversal com carreadores de variantes no gene TTR sem sintomas cardiovasculares e sem alterações nos parâmetros da eletrocardiografia ou do ecocardiograma convencional. Todos os pacientes foram submetidos à cintilografia com pirofosfato e à ecocardiografia com análise de *strain* . O envolvimento cardíaco subclínico, definido como um escore de Perugini ≥ 2, razão Coração (C)/ Hemitórax Contralateral (CL) ≥ 1,5 em uma hora, C/CL ≥ 1,3 na terceira hora, ou um *strain* longitudinal global (SGL) ≤ −17%. Realizadas análises descritiva e analítica, e aplicados o teste exato de Fisher e o teste de Mann-Whitney. Um valor de p<0,05 foi considerado significativo.

**Resultados:**

Os 23 pacientes avaliados apresentavam uma idade mediana de 51 (37-57) anos, 15 (65,2%) eram do sexo feminino, 12 (52,2%) eram pardos, nove (39,1%) apresentavam hipertensão arterial sistêmica, e nove (39,1%) tinham um diagnóstico prévio de PAF. Dos nove pacientes com PAF, oito (34,8%) usavam tafamidis. As variantes genéticas identificadas foram Val142IIe, Val50Met e IIe127Val. O valor mediano do SGL foi −19% (-16% – −20%). Dos 23 pacientes, nove (39,1%; 95% CI = 29–49%) preencheram os critérios de envolvimento cardíaco, seis (26%) somente pelo critério do SGL. Não houve associação entre PAF e um carreador assintomático avaliado por ecocardiograma com análise de *strain* e pela cintilografia com pirofostato (p=0,19). A prevalência de hipertensão arterial sistêmica, diabetes mellitus, dislipidemia, tabagismo e SGL reduzido não foi diferente entre os grupos. A velocidade da onda e’ septal foi a única variável que apresentou diferença significativa entre os indivíduos com e sem SGL reduzido, com uma área sob a curva ROC de 0,80 (IC95% = 0,61–0,98, p = 0,027). A melhor acurácia diagnóstica foi alcançada com uma velocidade e’ septal ≤ 8,5 cm/s. Não houve associação entre o tipo de variante genética e o envolvimento cardíaco pré-clínico, nem entre o uso de tafamidis e este mesmo envolvimento (37,5% versus 40,0%, p = 0,90).

**Conclusão:**

O envolvimento cardíaco subclínico foi frequente em uma amostra de carreadores da variante genética do gene TTR. Um valor do SGL reduzido foi o achado mais comum. Não houve associação entre a presença de polineuropatia amiloidótica e o envolvimento subclínico. O tipo de variante genética não foi associado com envolvimento cardíaco precoce. Nesta amostra, o uso de tafamidis (20mg/dia) não foi associado com uma menor prevalência de envolvimento cardíaco subclínico.

## Introdução

Amiloidose por transtirretina (ATTR) é uma doença infiltrativa e grave causada pela deposição anormal de proteína que pode acometer diversos órgãos e sistemas, principalmente o coração e o sistema nervoso periférico e autonômico.^1 – 3^ Existem dois subtipos de ATTR, a ATTR selvagem e amiloidose por transtirretina hereditária (ATTRh). A ATTRh está relacionada a uma variante genética específica de uma proteína chamada de transtirretina (TTR), com transmissão autossômica dominante. Trata-se de uma doença progressiva e degenerativa, que ocorre pela deposição insidiosa da proteína TTR no espaço extracelular com o espectro fenotípico amplo.^2^ Na ATTRh, foco dessa pesquisa, os portadores da variante genética podem evoluir durante anos de forma assintomática. Uma parcela apresenta-se com sua forma neurológica, a Polineuropatia Amiloidótica Familiar (PAF) ou um fenótipo cardiológico, a cardiomiopatia por ATTR (CM ATTR). Outros, no entanto, manifestam-se através de um fenótipo misto de envolvimento cardíaco e do sistema nervoso periférico e autônomo.^3 , 4^

O acometimento do sistema cardiovascular pode manifestar-se como uma cardiomiopatia restritiva com sinais e sintomas de uma clássica insuficiência cardíaca (IC) com ou sem fração de ejeção reduzida.^1 – 3 , 5 , 6^ Bloqueios atrioventriculares, bloqueios de ramo, arritmias atriais e ventriculares, aumento dos átrios e "hipertrofia" ventricular esquerda e, menos frequentemente direita, são as principais manifestações clínicas. Até recentemente esta entidade clínica não possuía tratamento farmacológico específico, o que inexoravelmente levava estes pacientes a um prognóstico muito desfavorável.^1 – 3^

Em um artigo publicado em 2018 e em 2021 (sua extensão), ficou demonstrada a eficácia e segurança da droga tafamidis, um estabilizador da proteína TTR, com relevante tamanho de efeito em reduzir mortalidade total e de hospitalizações por IC associado à Amiloidose Cardíaca (AC).^5^ O envolvimento cardíaco é o principal marcador de mal prognóstico com sobrevida média de 2-4 anos se não tratado.^1 – 3^ Dessa forma, a identificação precoce da infiltração amiloide no miocárdio, com posterior acompanhamento clínico rigoroso, e foco na vigilância para o surgimento de sinais e sintomas se justifica na atualidade. Ferramentas acuradas para este fim ainda não estão estabelecidas na literatura e, diante dessa lacuna no conhecimento atual, desenvolvemos esta pesquisa. O seu objetivo primário foi o de avaliar a prevalência do envolvimento cardíaco subclínico em uma amostra de pacientes portadores da variante genética no gene TTR, com base no PYP e pela análise do strain miocárdico. Comparar os achados entre o PYP e o strain, avaliar a associação entre PAF e o envolvimento cardiológico subclínco e analisar se há associação entre alguma variante genética específica e a detecção de envolvimento cardíaco, foram então definidos como sendo os objetivos secundários.

## Métodos

Estudo descritivo e analítico, corte transversal conduzido em um centro especializado em tratamento de ATTR na Escola Bahiana de Medicina e Saúde Pública (EBMSP), em Salvador – Bahia – Brasil. O estudo foi aprovado pelo comitê de ética em pesquisa da EBMSP, cumprindo os pré-requisitos das leis nacionais (Resolução 466/2012) e internacionais para condução de pesquisa em seres humanos. Todos os participantes leram, concordaram e assinaram o TCLE específico do estudo.

### Desenho de estudo

Amostra de conveniência onde todos os participantes do estudo eram acompanhados no ambulatório de doenças raras da EBMSP, com diagnóstico de PAF ou por serem familiares do caso índice da forma neurológica da doença. A realização da pesquisa da variante genética da TTR a partir da coleta de saliva era uma opção pessoal e livre, oferecida aos maiores de 18 anos, realizada após aconselhamento genético e com disponibilidade de suporte psicológico gratuito. O diagnóstico de PAF era realizado por dois neurologistas com expertise em PAF, a partir da história clínica, história familiar de PAF, exame físico, utilização da eletroneuromiografia e biópsia de glândula salivar, quando indicada.

### Pacientes

Os participantes elegíveis para a pesquisa foram aqueles com PAF e os carreadores da variante do gene TTR, ambos os grupos assintomáticos do ponto de vista cardiovascular.

#### Critérios de inclusão

Pacientes maiores de 18 anos, assintomáticos do ponto de vista cardiovascular e com a variante do gene da TTR identificados pela da análise do DNA da saliva.

#### Critérios de exclusão

Pacientes ao ecocardiograma com dilatação atrial, remodelamento concêntrico, hipertrofia ventricular ou disfunção sistólica do ventrículo esquerdo, definida como fração de ejeção (FEVE) ≤ 50% pelo método de Simpson, foram excluídos. Não foram elegíveis também aqueles com disfunção diastólica do ventrículo esquerdo (maior do que grau I), janelas acústicas ecocardiográficas inadequadas, presença de bloqueio atrioventricular (BAV), bloqueio do ramo direito (BRD), bloqueio do ramo esquerdo (BRE), sobrecarga de câmaras, fibrilação atrial (FA) ao eletrocardiograma (ECG) de repouso e gestantes ou com suspeita de gravidez.

**Figura 1:**
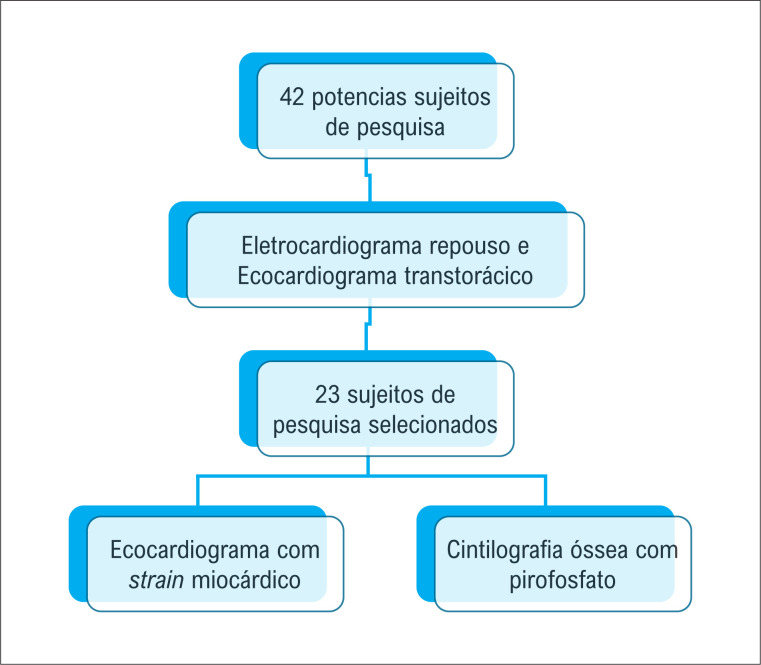
Fluxograma da seleção da amostra.

Todos passaram por uma consulta clínica prévia, preenchimento de uma ficha com dados antropométricos, demográficos, clínicos e caracterização das respectivas variantes genéticas. Após revisados quanto aos critérios de inclusão e exclusão, os pacientes eram encaminhados para a realização da cintilografia com PYP e o ecocardiograma com *strain.* Quarenta e dois potenciais candidatos a participar da pesquisa foram inicialmente submetidos a uma triagem por meio de revisão de prontuário quanto a sintomas cardiovasculares. Após isso, eram submetidos à realização de um ECG de repouso de 12 derivações e a um ecocardiograma transtorácico. Dos candidatos avaliados, 19 foram excluídos por serem sintomáticos, por possuírem achados no ECG de repouso ou limitações de janelas acústicas ao ecocardiograma transtorácico ( Figura 1 ).

Após isso, em um segundo dia, eram encaminhados para a realização do ecocardiograma com *speckle tracking* e para a cintilografia miocárdica com PYP.

### Protocolo da Cintilografia com pirofosfato (PYP)

Os participantes foram submetidos à cintilografia com PYP utilizando o equipamento da Siemens ( *Symbia Evo Excel* ) e seguindo-se os protocolos internacionais de investigação da AC.^7 , 8^ As imagens planares do tórax em projeções anterior e lateral esquerda eram adquiridas uma e três horas após administração venosa de 20mCi de PYP^99m^ Tc, seguidas de imagens tomográficas (SPECT) do miocárdio para afastar a presença de pool sanguíneo. As regiões de interesse foram definidas sobre a projeção do Coração (C) e no Hemitórax Contralateral (CL) para determinação da relação C/CL. Os critérios de positividade foram assim definidos: grau de captação do coração em relação aos arcos costais maior ou igual a 2 e/ou relação C/CL ≥ 1,5 na primeira hora ou ≥ 1,3 na terceira hora.^7 – 11^

### Protocolo do Ecocardiograma com *speckle tracking*

Os exames ecocardiográficos eram realizados em equipamento portátil da Philips, modeloCX 50, versão 5.0.2, sistema iE33 ( *Phillips Medical Systems* ), equipado com uma sonda setorial S 4-2. As imagens foram adquiridas por um investigador com experiência na realização da metodologia de avaliação da deformidade miocárdica ( *strain* ) de forma cega em relação aos resultados da cintilografia. Foi utilizada a quantificação automatizada de movimento cardíaco (aCMQ - *Automated Cardiac Motion Quantification* ) e a ferramenta QLAB do aparelho CX 50 para calcular a deformação longitudinal global do ventrículo esquerdo. A aquisição e interpretação das imagens seguiram as recomendações da *American Society of Echocardiography* .^12^ As janelas de duas câmaras, três câmaras e quatro câmaras foram usadas para medir o *strain* global longitudinal (SGL) e avaliar a presença ou não da imagem conhecida como *apical sparing.* O valor considerado normal para o SGL foi de ≥ − 18%.^12 , 13^

### Envolvimento cardíaco subclínico

Envolvimento subclínico foi definido por: escore visual (semi-quantitativo) ≥ 2 e/ou relação C/CL ≥ 1,5 em 1 hora ou ≥ 1,3 na terceira após a infusão do radiotraçador (escore quantitativo) e /ou SGL ≤ −17% pelo ecocardiograma transtorácico.

### Análise estatística

Os dados foram coletados e tabulados. O programa *Statistical Package for the Social Sciences* (SPSS), versão 25.0 (IBM) foi usado para as análises. As variáveis categóricas foram descritas por percentual (proporção) e as variáveis contínuas por mediana e intervalo interquartil (não paramétricas). Dados categóricos foram comparados pelo teste Exato de Fisher e os contínuos pelo Mann Whitney (não paramétricas). O teste de Shapiro-Wilk foi usado para verificar a normalidade da distribuição dos dados. Uma curva ROC foi utilizada para comparar a acurácia diagnóstica entre uma variável numérica e uma categórica. Um valor de p < 0,05 foi utilizado como parâmetro de significância estatística em todas as análises.

## Resultados

A amostra consistiu em 23 participantes, todos assintomáticos do ponto de vista cardiovascular ( Tabela 1 ).

**Tabela 1:** Características demográficas e ecocardiográficas dos participantes

Características		Tamanho amostral (n=23)
**Idade (anos)**		51 (IQ * 37-57)
**Sexo, n° (%)** **Raça, n° (%)**		Feminino, 15 (65)
		Parda, 12 (52,2)
		Negra, 7 (30,4)
		Branca, 4(17,4)
**Tipo de mutação, n° (%)**		Val142IIe, 9 (43)
		Val50met, 8 (35)
		IIe127Val, 5 (22)
**Portadores assintomáticos, n° (%)**		**14 (61)**
	PAF, n° (%)	9 (39,1)
	HAS, n° (%)	9 (39,1)
	DM, n° (%)	4 (17,4)
	DLP, n° (%)	6 (26)
	Tabagismo atual, n° (%)	2 (8,7)
	Uso de tafamidis, n° (%)	8 (34,8)
**FE (%)**		**69 (IQ 66 - 72)**
	Diâmetro diastólico do VE (mm)	44 (IQ 40 - 48)
	Diâmetro sistólico do VE (mm)	27(IQ 26 - 30)
	Septo interventricular (mm)	9 (IQ 8 - 10)
	Parede posterior (mm)	8 (IQ 8 - 9)
**Índice de massa do VE (g/m^2^)**		**72 (IQ 62 - 81)**
	Volume atrial esquerdo (ml/m^2^)	29 (IQ 26 - 31)
	Velocidade da onda E mitral (cm/s)	80 (IQ 61 – 92)
	Velocidade da onda A mitral (cm/s)	68 (IQ 59 – 80)
	Relação E/A	1,0 (IQ 0,9 – 1,6)
	Velocidade da onda e septal do Doppler tecidual (cm/s)	9,0 (IQ 8 – 10)
	Velocidade da onda e lateral do Doppler tecidual (cm/s)	11 (IQ 10 – 12)
	Relação E/e’ média	8,1 (IQ 6,8 – 9,0)
**Strain global longitudinal (%)**		- 19 (IQ – 16% a – 20%)

* IQ: Intervalo interquartil; FAP: polineuropatia amiloidótica familiar; HAS: hipertensão arterial sistêmica; DM: diabetes mellitus; DLP: dislipidemia; FE: fração de ejeção; VE: ventrículo esquerdo.

**Figura 2:**
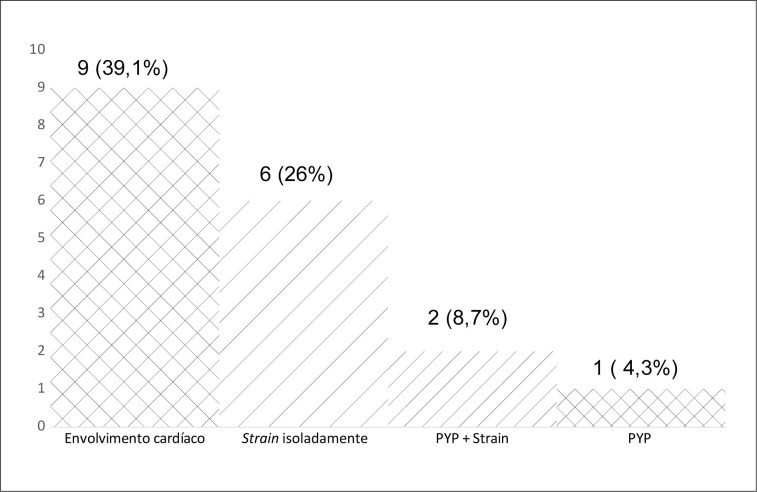
Prevalência de envolvimento cardíaco subclínico por método diagnóstico: cintilografia com pirofosfato e ecocardiograma com *strain* .

Com base nos resultados da cintilografia com PYP e do ecocardiograma com *strain* , 14 participantes (60,9%) não demonstraram alterações nos exames cardiológicos. Nove (39,1%; 95%IC 29% - 49%) apresentaram critérios de envolvimento cardíaco, sendo seis (26%) com critério pelo *strain* isoladamente, um (4,3%) com PYP isoladamente e dois (8,7%) com alteração tanto ao PYP quanto ao *strain* ( Figura 2 ).

A presença de *blood pool* foi constatada em três participantes (SPECT realizado na primeira e terceira horas após a injeção do PYP).

**Figura 3:**
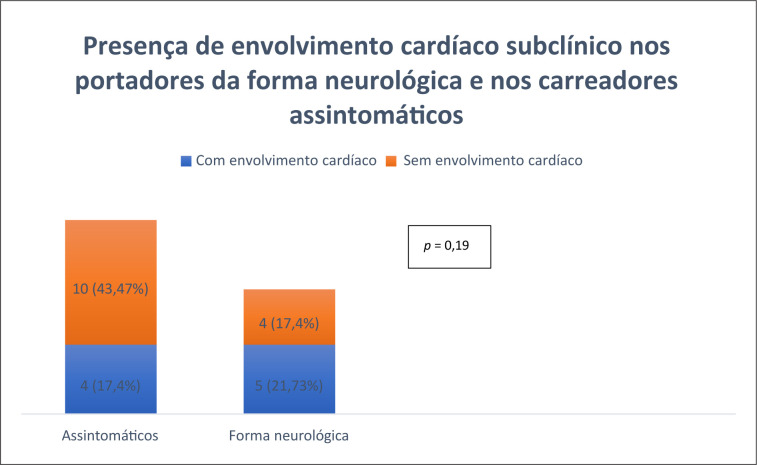
Gráfico de barras representando a presença de envolvimento cardíaco nos assintomáticos e nos portadores de Polineuropatia Amiloidótica Familiar, teste exato de Fisher.

Não houve associação entre ter PAF e ser um carreador assintomático quando se avaliou a presença ou não de envolvimento cardíaco pelo ecocardiograma com *strain* ou pela cintilografia com PYP (p = 0,19). Dentre os 14 carreadores assintomáticos, quatro (17,4%) tinham evidências de envolvimento cardíaco pré-clínico, enquanto dentre os nove pacientes com PAF, cinco (21,7%) apresentavam achados sugestivos de comprometimento subclínico ( Figura 3 ).

Não foi encontrada diferença na prevalência de hipertensão arterial sistêmica (HAS), diabetes mellitus (DM), dislipidemia (DLP) e tabagismo quando se comparou os pacientes com e sem *strain* reduzido ( Tabela 2 ).

**Tabela 2:** Características dos participantes por valor do strain global longitudinal (SGL)

Características		SGL ≥ −18%	SGL ≤ −17%	Valor de p
HAS, n° (%)		7 (43,8)	2 (25,0)	0,41
DM, n° (%)		4 (25,0)	0	0,27
DLP, n° (%)		6 (37,5)	0	0,12
Tabagismo ativo, n° (%)		2 (12,5)	0	0,5
* **Parâmetros da função diastólica**				
	Volume do átrio esquerdo (mL/m^2^)	29 (26-31)	29 (28-31)	0,62
	Velocidade E (cm/s)	84,5 (64,2-97,2)	68,0 (61-86)	0,37
	Velocidade A (cm/s)	67,5 (59-79,5)	69,0 (59-92)	0,82
	Relação E/A	1,1 (0,96-1,77)	1,0 (0,87-1,19)	0,37
	Velocidade da onda e' septal (cm/s)	9,0 (8,3-11)	8,0 (8-8)	**0,027**
	Velocidade da onda e' lateral (cm/s)	11 (10-13,5)	10 (10-11)	0,10
	Relação E/e' média	8,1 (6,8-9,0)	7,4(6,8-9,7)	0,99

* As variáveis da função diastólica foram descritas em mediana e intervalo interquartil; diferença entre os grupos foi analisada usando o teste exato de Fisher para as variáveis categóricas e o teste de Mann-Whitney para as contínuas; HAS: hipertensão arterial sistêmica, DM: Diabetes Mellitus; DLP: Dislipidemia.

As variáveis determinantes da função diastólica (velocidade da onda E e A do Doppler mitral, relação onda E/A, onda e’ do Doppler tecidual septal e lateral e valor médio da E/e’) foram comparadas entre os pacientes com e sem strain reduzido ( Tabela 2 ).

**Figura 4:**
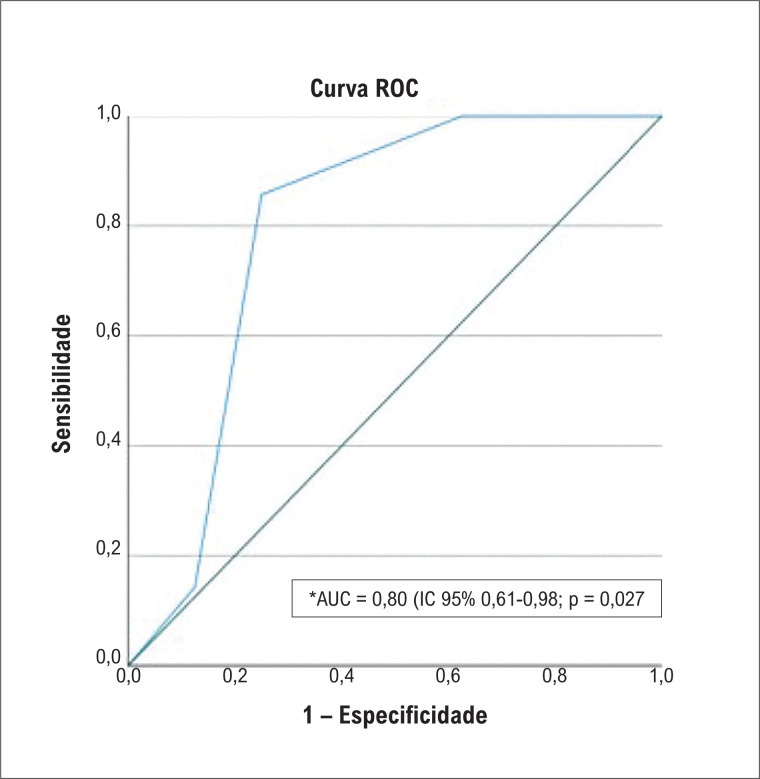
Curva ROC representando a acurácia diagnóstica entre a velocidade da onda e'septal e *strain* miocárdico. *AUC: area under the curve; ROC: Receiver Operating Characteristic.

A única variável que diferiu de forma significativa entre os grupos foi a velocidade da onda e’ septal do Doppler tecidual, com uma área abaixo da curva ROC de 0,80 (IC 95% 0,61 – 0,98; p = 0,027). Uma velocidade menor ou igual a 8,5 cm/s foi a que atingiu a melhor acurácia diagnóstica para predizer a presença de um *strain* reduzido ( Figura 4 ).

**Figura 5:**
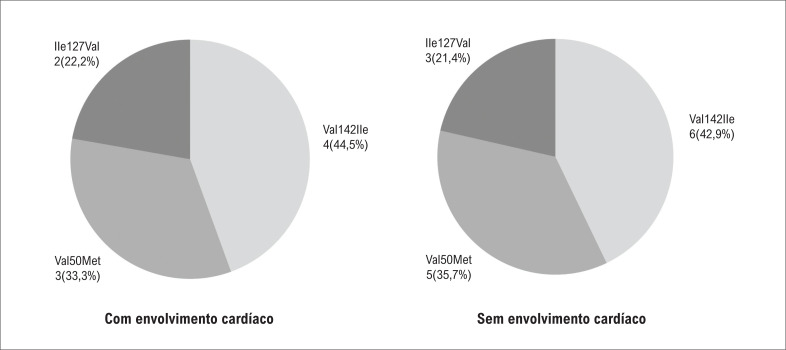
Distribuição das variantes genéticas nos pacientes com envolvimento cardíaco e sem envolvimento cardíaco. Teste Exato de Fischer.

**Figura 6:**
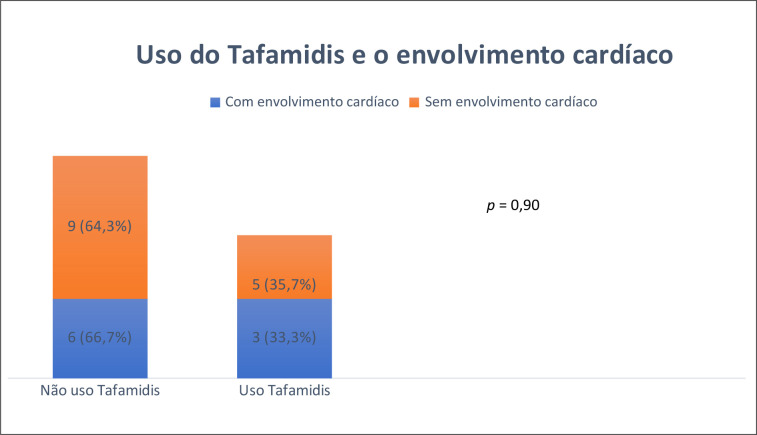
Frequência da presença de envolvimento cardíaco em pacientes com e sem uso de tafamidis. Teste Exato de Fischer.

A associação entre o tipo de variante genética e a presença de envolvimento cardíaco pré-clínico não foi encontrada ( Figura 5 ), assim como não houve associação entre o uso de tafamidis e um menor grau de envolvimento cardíaco (p = 0,90) ( Figura 6 ).

## Discussão

O envolvimento cardíaco subclínico foi verificado em 39% (n=9) do total dos participantes. Seis sujeitos da pesquisa apresentaram o valor do SGL alterado de forma isolada, dois preencheram os critérios cintilográficos e ecocardiográficos e um participante exclusivamente pelo critério do PYP ( Figura Central ). Em três participantes o *blood pool* , que é sabidamente uma das causas de falso-positivo,^7 – 9^ foi constatado após a realização do *SPECT* .

**Figure:**
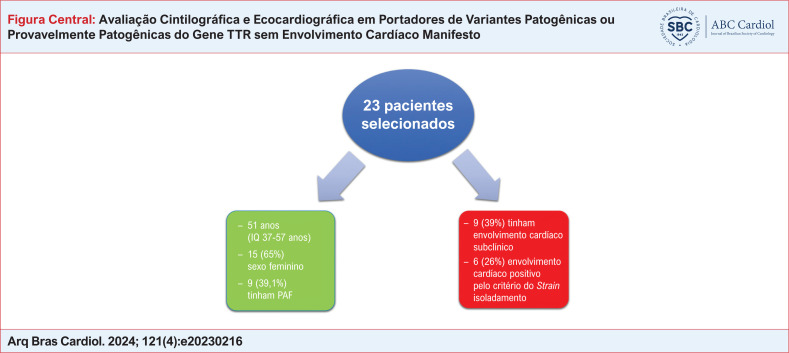


Quando se comparou a presença de envolvimento cardíaco entre os pacientes com PAF (n=5) e os portadores assintomáticos (n=4), não houve associação (p = 0,19). Foram identificadas nessa pesquisa três tipos de variantes genéticas, uma delas classicamente mais relacionada ao polo cardíaco de manifestação clínica (Val142IIe).^3 , 14 – 16^ Quando da análise destas variantes genéticas com a variável envolvimento cardíaco, não foi identificado diferença entre elas. O sexo feminino foi o mais prevalente (65%) na nossa amostragem; este achado pode ter alguma relevância, visto que classicamente a TTR variante ou hereditária envolve predominantemente o sexo masculino.^3 , 17 – 20^ O parâmetro ecocardiográfico da função diastólica que demostrou associação positiva com *strain* miocárdico alterado foi a velocidade da onda e’ septal do Doppler tecidual. O valor ≤ 8,5 cm/s foi o que atingiu a melhor acurácia, com uma área abaixo da curva ROC de 0,80 (IC 95% 0,61- 0,98) valor de p = 0,027. Este é um dado ainda exploratório, valendo destacar que < 7cm/s é considerado reduzido para a população em geral, mas pode sugerir que um valor ≤ 8,5cm/s da onda e’ septal esteja associado a um envolvimento cardíaco pré-sintomático, podendo ser útil, por exemplo, para triagem de pacientes quando o *strain* não for disponível. Por ser uma medida ecocardiográfica feita de forma rotineira, rápida e fácil, acreditamos que esta informação possa ter, em um futuro próximo, potencial aplicabilidade prática.

Nesta amostra não houve diferença na prevalência de HAS, DM, DLP e tabagismo, quando se comparou os pacientes com e sem *strain* reduzido. Não houve associação entre o tipo de variante genética e a presença de envolvimento cardíaco pré-clínico, assim como não houve associação entre o uso de tafamidis 20mg/dia e uma menor prevalência de envolvimento cardíaco (37,5% versus 40,0%, p = 0,90). É importante ressaltar que o período de uso de Tafamidis foi de apenas 6 meses, podendo não representar um tempo adequado para a demonstração de algum benefício do ponto de vista cardiovascular.

Dentre os critérios pré-estabelecidos para a identificação do envolvimento miocárdico subclínico, o valor do SGL foi o parâmetro mais comumente alterado, no entanto a imagem clássica em AC conhecida como *apical sparing* não foi vista em nenhum dos sujeitos da pesquisa.

A ecocardiografia com *strain* é sabidamente um método sensível para o diagnóstico de acometimento cardíaco pré-clínico.^13^ A técnica alicerça-se como ferramenta de análise da função sistólica, notadamente em cardio-oncologia,^14^ porém com dados menos robustos em AC-ATTR.^2 , 3^ Estudos da literatura vem tentando demonstrar a importância do diagnóstico precoce na AC utilizando-se o próprio *strain* longitudinal e o circunferencial, além da cintilografia com marcadores ósseos e a ressonância cardíaca pelo mapa T1 e pelo volume extracelular.^1 , 2^

A história natural dos portadores assintomáticos da variante genética da TTR e daqueles com a forma "exclusivamente" neurológica ainda não está completamente definida.^1^ Tipos de variantes genéticas, graus de penetrância, expressividade e fatores ambientais interagem ativamente nesse processo.^1 , 2^ O exame não invasivo mais sensível para a pesquisa de envolvimento subclínico é um campo com opiniões divergentes, de certa forma ainda incerto e consequentemente de intensa pesquisa e interesse. Biomarcadores como troponina e BNP, eletrocardiograma de repouso e ecocardiograma convencional são ferramentas pouco acuradas nesse perfil de pacientes.^1 , 2 , 15^ Em estudo publicado recentemente utilizou-se a dosagem da proteína ligada ao retinol 4 (RBP4 – *retinol binding protein* 4) e a dosagem sérica da TTR mal dobrada, na tentativa de discriminar o "doente" subclínico.^1^ Já Quarta et al.^16^ demonstraram que existiam alterações ecocardiográficas mais prevalentes nos carreadores da variante genética Val142Ie, como aumento da espessura da parede posterior do ventrículo esquerdo, relação E/e’ e anormalidade do valor do SGL.^16^ Sinha et al.,^17^ em 2020, mostraram um valor do *strain* circunferencial sistólico reduzido nos portadores da variante genética Val142Ie em relação aos controles. No entanto, ambos os estudos tiveram com importantes limitações a não realização de outras modalidades para o diagnóstico desse comprometimento subclínico, como por exemplo biópsia, ressonância cardíaca ou cintilografia com PYP.^1 , 16 , 17^ Em 2017, Haq et al.^18^ avaliaram 40 pacientes, com objetivo de determinar o valor do depósito de PYP como marcador muito precoce de depósito amiloide no coração de indivíduos com genótipo positivo, mas sem IC. Além do PYP, foram realizadas a dosagem de troponina I e BNP e realizado o ecocardiograma transtorácico. Na análise dos 12 carreadores da variante genética, 10 (84%) tinham algum grau de captação e sete casos (58%) demostraram grau 2 ou 3 de captação do PYP. A relação C/CL nos assintomáticos foi 1,5 +/- 0,4 versus 1,2 +/- 0,1 nos com ICFEp não amiloide, com valor de p=0,02. Os autores concluíram que a captação anormal de PYP pode ser a primeira manifestação detectável dos carreadores assintomáticos, antes mesmo dos biomarcadores, do eletrocardiograma e ecocardiograma. Nesta publicação, a ressonância cardíaca e o ecocardiograma com *strain* não foram analisados.^18^

Em nossa pesquisa, tanto o PYP quanto o strain foram realizados, sendo então, talvez, uma abordagem mais acurada em relação as publicações anteriores. Dada a praticidade, segurança e não necessidade da utilização de radiação no exame ecocardiográfico com *strain* , a proposta de inserir este procedimento no diagnóstico dos assintomáticos do ponto de vista cardiovascular, parece-nos promissora. Apesar do PYP ter sido positivo em três participantes, a redução do SGL foi mais comumente observada. Estes dados agregam novas informações a respeito da utilidade e relevância do *strain* miocárdico no manejo desse grupo de pacientes, fazendo com que a deformação miocárdica possa ser vista como mais uma, ou quem sabe, a mais sensível ferramenta no estudo das pessoas sem sintomas cardiovasculares manifestos.

É importante salientar também que em três pacientes completamente assintomáticos, com eletrocardiograma e ecocardiograma normais, a cintilografia com PYP mostrou-se alterada, sugerindo a existência de depósitos de material amiloide no coração. Tomando como base a prática clínica atual, carreadores assintomáticos e até os com PAF não são investigados rotineiramente quanto à presença de deposição de amiloide no miocárdio, o que possivelmente precisa ser revisto. Na nossa amostragem, no entanto, nos portadores de PAF, o envolvimento cadíaco subclínico concomitante não foi observado. Isto pode ser justificado pelo pouco tempo, na nossa população, para que tenha ocorrido a infiltração da proteína TTR no miocárdio, ou até mesmo porque trata-se de doenças distintas, o que vai de encontro a outros dados na literatura.^18^

À medida que mais se investiga, mais pessoas são diagnosticadas e mais familiares do caso índice apresentam-se nos consultórios médicos com suas angústias e questionamentos.^1 – 4 , 19^ Assim, é fundamental que tenhamos uma resposta de como segui-los e quais são os exames de *screening* mais sensíveis. Podemos dizer então que este estudo coloca-se na vanguarda deste tema.

Os portadores da variante genética da TTR estão sob risco de desenvolver em algum momento da vida um quadro de IC.^1 – 4^ Comportamentos proativos, respaldados em ciência de boa qualidade podem modificar a história natural desta doença de curso progressivo e muitas vezes inexorável,^1 – 4 , 19^ e é justamente essa a nossa proposta e ponto de maior destaque.

Apresentamos aqui um trabalho que tem como perspectiva o potencial de ajudar a discriminar indivíduos efetivamente sem envolvimento miocárdico subclínico daqueles com algum grau de depósito amiloide no coração. Com isso um acompanhamento mais regular e focado no aparecimento dos sinais e sintomas desta entidade clínica poderá ser instituído. No entanto, ainda hoje não sabemos com precisão qual o exame não invasivo mais sensível para diagnosticar esse envolvimento, e mais, diante dos resultados, qual a melhor conduta a ser seguida.^1 – 4^ Apesar do grande avanço na consolidação do diagnóstico não invasivo da AC-ATTR,^7 – 11^ o capítulo final da melhor abordagem quando estamos diante dos portadores assintomáticos ainda não foi devidamente escrito.^1 – 4^ Estudos maiores devem ser realizados para que SGL e/ou a cintilografia com PYP possam se consolidar, assumindo uma posição de protagonismo no fluxo diagnóstico dos carreadores assintomáticos e também naqueles com a forma neurológica da doença.

O ecocardiograma com *strain* necessita, assim como qualquer técnica ecocardiográfica, de uma adequada janela acústica, além de ser examinador e equipamento dependentes.^12 , 13^ É conhecida também e considerada um fator limitante do método e por consequente deste estudo, a sua variabilidade entre observadores e intraobservador,^12 , 13^ o que de certa forma reduz a precisão e a reprodutibilidade desta ferramenta. Apesar da experiência na realização do método, este procedimento foi realizado por um único ecocardiografista, o que pode ser considerado uma limitação em sua metodologia. O número de 23 participantes sinaliza para que tenhamos cuidado em relação a extrapolações dos seus resultados finais. Em amostras pequenas, o acaso encontra-se muito mais presente e o risco de conclusões precipitadas estão intrinsicamente relacionadas ao erro tipo 1. Vale salientar que, apesar da limitação do número de participantes no estudo, a amiloidose por TTR é uma doença rara ou infrequente, gerando inerentes limitações amostrais.

## Conclusão

Em uma amostra de pacientes portadores de variantes genéticas da TTR, assintomáticos do ponto de vista cardiovascular e com exames cardiológicos de triagem sem alterações, o envolvimento cardíaco subclínico foi frequente. A redução do valor do SGL do ventrículo esquerdo foi o parâmetro mais comumente verificado. Não houve relação entre a presença de PAF e o envolvimento cardíaco subclínico. O tipo de variante genética não se associou a um envolvimento cardíaco precoce e, na amostra estudada, o uso prévio de tafamidis não se associou com uma menor prevalência de envolvimento cardíaco subclínico.

Estudos maiores devem ser realizados para que o *strain* global e a cintilografia com PYP possam assumir uma posição de protagonismo na investigação diagnóstica dos carreadores assintomáticos e naqueles com a forma neurológica da doença.

## References

[1] Griffin JM, Rosenthal JL, Grodin JL, Maurer MS, Grogan M, Cheng RK (2021). ATTR Amyloidosis: Current and Emerging Management Strategies: JACC: CardioOncology State-of-the-Art Review. JACC CardioOncol.

[2] Maurer MS, Bokhari S, Damy T, Dorbala S, Drachman BM, Fontana M (2019). Expert Consensus Recommendations for the Suspicion and Diagnosis of Transthyretin Cardiac Amyloidosis. Circ Heart Fail.

[3] Ruberg FL, Grogan M, Hanna M, Kelly JW, Maurer MS (2019). Transthyretin Amyloid Cardiomyopathy: JACC State-of-the-Art Review. J Am Coll Cardiol.

[4] Pinto MV, Barreira AA, Bulle AS, Freitas MRG, França MC, Gondim FAA (2018). Brazilian Consensus for Diagnosis, Management and Treatment of Transthyretin Familial Amyloid Polyneuropathy. Arq Neuropsiquiatr.

[5] Maurer MS, Schwartz JH, Gundapaneni B, Elliott PM, Merlini G, Waddington-Cruz M (2018). Tafamidis Treatment for Patients with Transthyretin Amyloid Cardiomyopathy. N Engl J Med.

[6] González-López E, Gallego-Delgado M, Guzzo-Merello G, Haro-Del Moral FJ, Cobo-Marcos M, Robles C (2015). Wild-type Transthyretin Amyloidosis as a Cause of Heart Failure with Preserved Ejection Fraction. Eur Heart J.

[7] Dorbala S, Ando Y, Bokhari S, Dispenzieri A, Falk RH, Ferrari VA (2019). ASNC/AHA/ASE/EANM/HFSA/ISA/SCMR/SNMMI Expert Consensus Recommendations for Multimodality Imaging in Cardiac Amyloidosis: Part 1 of 2-evidence Base and Standardized Methods of Imaging. J Nucl Cardiol.

[8] Dorbala S, Ando Y, Bokhari S, Dispenzieri A, Falk RH, Ferrari VA (2019). ASNC/AHA/ASE/EANM/HFSA/ISA/SCMR/SNMMI Expert Consensus Recommendations for Multimodality Imaging in Cardiac Amyloidosis: Part 2 of 2-Diagnostic Criteria and Appropriate Utilization. J Card Fail.

[9] Singh V, Falk R, Di Carli MF, Kijewski M, Rapezzi C, Dorbala S (2019). State-of-the-art Radionuclide Imaging in Cardiac Transthyretin Amyloidosis. J Nucl Cardiol.

[10] Perugini E, Guidalotti PL, Salvi F, Cooke RM, Pettinato C, Riva L (2005). Noninvasive Etiologic Diagnosis of Cardiac Amyloidosis Using 99mTc-3,3-diphosphono-1,2-propanodicarboxylic acid Scintigraphy. J Am Coll Cardiol.

[11] Gillmore JD, Maurer MS, Falk RH, Merlini G, Damy T, Dispenzieri A (2016). Nonbiopsy Diagnosis of Cardiac Transthyretin Amyloidosis. Circulation.

[12] Voigt JU, Pedrizzetti G, Lysyansky P, Marwick TH, Houle H, Baumann R (2015). Definitions for a Common Standard for 2D Speckle Tracking Echocardiography: Consensus Document of the EACVI/ASE/Industry Task Force to Standardize Deformation Imaging. J Am Soc Echocardiogr.

[13] Potter E, Marwick TH (2018). Assessment of Left Ventricular Function by Echocardiography: The Case for Routinely Adding Global Longitudinal Strain to Ejection Fraction. JACC Cardiovasc Imaging.

[14] Liu J, Banchs J, Mousavi N, Plana JC, Scherrer-Crosbie M, Thavendiranathan P (2018). Contemporary Role of Echocardiography for Clinical Decision Making in Patients During and After Cancer Therapy. JACC Cardiovasc Imaging.

[15] Pagourelias ED, Mirea O, Duchenne J, Van Cleemput J, Delforge M, Bogaert J (2017). Echo Parameters for Differential Diagnosis in Cardiac Amyloidosis: A Head-to-Head Comparison of Deformation and Nondeformation Parameters. Circ Cardiovasc Imaging.

[16] Quarta CC, Buxbaum JN, Shah AM, Falk RH, Claggett B, Kitzman DW (2015). The Amyloidogenic V122I Transthyretin Variant in Elderly Black Americans. N Engl J Med.

[17] Sinha A, Zheng Y, Nannini D, Qu Y, Hou L, Shah SJ (2020). Association of the V122I Transthyretin Amyloidosis Genetic Variant With Cardiac Structure and Function in Middle-aged Black Adults: Coronary Artery Risk Development in Young Adults (CARDIA) Study. JAMA Cardiol.

[18] Haq M, Pawar S, Berk JL, Miller EJ, Ruberg FL (2017). Can 99mTc-Pyrophosphate Aid in Early Detection of Cardiac Involvement in Asymptomatic Variant TTR Amyloidosis?. JACC Cardiovasc Imaging.

[19] Adams D, Koike H, Slama M, Coelho T (2019). Hereditary Transthyretin Amyloidosis: A Model of Medical Progress for a Fatal Disease. Nat Rev Neurol.

[20] Lane T, Fontana M, Martinez-Naharro A, Quarta CC, Whelan CJ, Petrie A (2019). Natural History, Quality of Life, and Outcome in Cardiac Transthyretin Amyloidosis. Circulation.

