# Supplementary material for: Avaliação Cintilográfica e Ecocardiográfica em Portadores de Variantes Patogênicas ou Provavelmente Patogênicas do Gene TTR sem Envolvimento Cardíaco Manifesto
Source: Arq Bras Cardiol. 2024 May 7;121(4):e20230216. [Article in Portuguese] doi: 10.36660/abc.20230216 (PMC11081098; doi:10.36660/abc.20230216)
Supplement: Translation

# Translation: Scintigraphic and Echocardiographic Study of Patients with Pathogenic or Probably Pathogenic Variants of the TTR Gene without Overt Cardiac Involvement

**DOI:** 10.36660/abc.20230216i

**Authors:** Tonnison de Oliveira Silva, Eduardo Sahade Darzé, Marcela Machado Costa, Luiz José Junior, Antonio A. B. Ximenes, Fábio Fernandes, Mário de Seixas Rocha, Marcia M. Noya-Rabelo, Luiz Eduardo Fonteles Ritt

**Affiliations:** 1 Salvador BA Brazil Hospital Cardio Pulmonar - Instituto D'Or de Ensino e Pesquisa – IDOR, Salvador, BA – Brazil; 2 Salvador BA Brazil Escola Bahiana de Medicina e Súde Pública, Salvador, BA – Brazil; 3 São Paulo SP Brazil Instituto do Coração do Hospital das Clínicas da Faculdade de Medicina da Universidade de São Paulo, São Paulo, SP – Brazil; 4 Salvador BA Brazil Hospital São Rafael - Instituto D'Or de Ensino e Pesquisa – IDOR, Salvador, BA – Brazil

**Keywords:** Amiloidose, Familial Amyloid Neuropathies, Echocardiography, Radionuclide Imaging

## Abstract

**Background:**

Transthyretin amyloidosis (ATTR) is an infiltrative disease caused by abnormal protein deposition mainly in the heart and peripheral nervous system. When it affects the heart, the disease presents as restrictive cardiomyopathy; when it affects the peripheral and autonomic nervous system, it manifests as polyneuropathy, and is called familial amyloid polyneuropathy (FAP). There are two ATTR subtypes: wild-type ATTR, where there is no mutation, and mutant ATTR (ATTRm), which is characterized by a mutation in the gene encoding the transthyretin protein (TTR). In both subtypes, cardiac involvement is the major marker of poor prognosis.

**Objectives:**

To assess the prevalence of subclinical cardiac involvement in a sample of patients with TTR gene mutation by using pyrophosphate scintigraphy and strain echocardiography; to compare scintigraphy and strain findings; to evaluate the association between neurological manifestations (FAP) and subclinical cardiac involvement; and to analyze whether there is an association between any specific mutation and cardiac involvement.

**Methods:**

This is a cross-sectional study with carriers of the TTR gene mutation, without cardiovascular symptoms or changes in electrocardiographic or conventional echocardiographic parameters. All patients underwent pyrophosphate scintigraphy and strain echocardiography. Subclinical cardiac involvement was defined as a Perugini score ≥ 2, heart-to-contralateral lung (H/CL) ratio ≥ 1.5 at 1 h, H/CL ≥1.3 at 3 h, or global longitudinal strain (GLS) ≤ −17%. Descriptive and analytical analyses were performed and Fisher's exact test and Mann–Whitney test were applied. A value of p < 0.05 was considered significant.

**Results:**

The 23 patients evaluated had a median age of 51 years (IQR 37–57 years), 15 (65.2%) were female, 12 (52.2%) were Pardo, nine (39.1%) had systemic arterial hypertension, and nine (39.1%) had a previous diagnosis of FAP. Of the nine patients with FAP, 8 (34.8%) were on tafamidis. The associated mutations were Val142IIe, Val50Met, and IIe127Val. The median GLS in the sample was −19% (−16% to −20%). Of the 23 patients, nine (39.1%; 95% CI = 29–49%) met criteria for cardiac involvement, six (26%) by the GLS-based criteria only. There was no association between having FAP and being an asymptomatic carrier, as assessed by strain echocardiography and pyrophosphate scintigraphy (p = 0.19). The prevalence of systemic arterial hypertension, diabetes mellitus, dyslipidemia, smoking, and reduced GLS did not differ between groups. Septal e’ wave velocity was the only variable that significantly differed between individuals with and without reduced GLS, with an area under the ROC curve of 0.80 (95% CI = 0.61–0.98, p = 0.027). The best diagnostic accuracy was achieved with a septal e’ velocity ≤ 8.5 cm/s. There was no association between mutation type and preclinical cardiac involvement, nor between tafamidis use and lower degree of cardiac involvement (37.5% versus 40.0%, p = 0.90).

**Conclusion:**

Subclinical cardiac involvement was common in a sample of TTR mutation carriers without cardiac involvement. Reduced left ventricular GLS was the most frequent finding. There was no association between the presence of amyloid polyneuropathy and subclinical cardiac involvement. Type of mutation was not associated with early cardiac involvement. In this sample, the use of tafamidis 20 mg/day was not associated with a lower prevalence of subclinical cardiac involvement.

## Introduction

Transthyretin amyloidosis (ATTR) is a severe infiltrative disease caused by abnormal protein deposition that may affect several organs and systems, especially the heart and the peripheral and autonomous nervous system.^1 – 3^There are two ATTR subtypes: a wild-type ATTR and hereditary ATTR amyloidosis (hATTR). hATTR is related to a specific protein, called transthyretin (TTR), and is transmitted in an autosomal dominant mode. It is a progressive degenerative disease that results from the gradual deposition of TTR in the extracellular space, with a wide phenotypical spectrum.^2^In hATTR, the focus of this study, the carriers of TTR mutation may be asymptomatic for years; a small proportion of patients develop the neurological disease, familial amyloidotic polyneuropathy (FAP), the cardiac phenotype (TTR amyloid cardiomyopathy), or a mixed form, with the involvement of both cardiovascular and (peripheral and autonomic) nervous system.^3 , 4^

When the cardiovascular system is affected, manifestations include restrictive cardiomyopathy with signs and symptoms of classic heart failure (HF) with or without reduced ejection fraction.^1 – 3 , 5 , 6^Atrioventricular blocks, branch blocks, atrial and ventricular arrhythmias, atrial enlargement, left ventricular "hypertrophy" and, less frequently, right ventricular hypertrophy are the main clinical manifestations. Until recently, this clinical entity did not have specific pharmacological treatment, which inevitably led to a very poor prognosis.^1 – 3^

In an article published in 2018 and in 2021 (its extension), the efficacy and safety of tafamidis, a TTR stabilizer, was demonstrated, with a significant size effect in reducing all-cause mortality and cardiovascular-related hospitalizations for HF associated with cardiac amyloidosis (CA).^5^Cardiac involvement is the main marker of poor prognosis, with a mean survival time of 2-4 years if untreated.^1 – 3^Thus, an early identification of myocardial amyloid infiltration, followed by a close clinical follow-up focusing on surveillance for signs and symptoms is currently justified. Accurate tools for this purpose have not been defined in the literature. Based on this gap in the current knowledge, the aims of the present study were to assess the prevalence of subclinical cardiac involvement in a sample of patients with TTR gene mutation by using pyrophosphate (PYP) scintigraphy and strain echocardiography; to compare scintigraphic and strain analysis findings; to evaluate the association between the presence of neurological manifestations (FAP) and subclinical cardiac involvement; and to evaluate whether there is an association between any specific mutation and the presence of cardiac involvement.

## Methods

This was a descriptive, analytical, cross-sectional study conducted in a referral center for ATTR treatment, located at Bahiana Medical and Public Health School (EBMSP) in Salvador, Brazil. The study was designed according to the Brazilian 466/2012 Resolution and the international criteria for research with human beings and was approved by the EBMSP ethics committee.

### Study design

This was a convenience sample of patients seen in the outpatient clinic for rare disease at EBMSP, with diagnosis of FAP, or family members of the index case of the neurological form of the disease. The screening for TTR mutation with saliva samples was offered for patients older than 18 years old, who were free to decide whether or not to participate. With their consent, the screening was performed after genetic counseling, and psychological services were also provided without charge. The diagnosis of FAP was made by two experienced neurologists using patients’ clinical history, family history of FAP, physical examination, electroneuromyography, and biopsy of the salivary glands when indicated.

### Patients

Patients with neurological manifestations of TTR mutation, without cardiovascular symptoms, and asymptomatic TTR mutation carriers with the disease were considered eligible for the study.

#### Inclusion criteria

Patients aged older than 18 years with TRR gene mutation (confirmed by salivary DNA analysis), and no cardiovascular symptoms.

#### Exclusion criteria

Patients with atrial dilatation, concentric remodeling, ventricular hypertrophy or left ventricular systolic dysfunction, defined as left ventricular ejection fraction (LVEF) ≤ 50% by the Simpson's method; patients with left ventricular diastolic dysfunction (diastolic dysfunction grade 1); patients with poor echocardiogram acoustic window; presence of atrioventricular block (AVB), right bundle branch block (RBBB), left bundle branch block (LBBB), and atrial fibrillation (AF) on resting electrocardiogram (ECG); and pregnancy or suspected pregnancy.

All patients attended a medical appointment, filled out a questionnaire containing questions on anthropometric, demographic, and clinical data, and characterization of respective mutations. After the inclusion and exclusion criteria were reviewed, patients were referred to PYP scintigraphy and strain echocardiogram. Forty-two potential candidates for the study were initially screened by medical record review for cardiovascular symptoms. Subsequently, the patients underwent a resting 12-lead ECG and a transthoracic echocardiogram. Nineteen candidates were excluded for being symptomatic, or for their ECG findings or due to technical limitations of transthoracic echocardiogram that met the exclusion criteria ( Figure 1 ).

**Figure 1:** Flowchart of patient selection.

On the second day, patients were referred to speckle tracking echocardiography and PYP myocardial scintigraphy.

### Pyrophosphate (PYP) scintigraphy protocol

Participants underwent PYP scintigraphy using a Simens Symbia Evo Excel, following the international protocols for CA.^7 , 8^Anterior and lateral planar images of the chest were acquired one and three hours after venous administration of 20 mCi of^99m^Tc-PYP, followed by SPECT computed tomography of the myocardium to rule out the presence of blood pool. Regions of interest were drawn over the heart (H) and contralateral lung (CL) to establish the H/CL ratio. Positivity criteria were defined as follows: cardiac uptake to costal arch uptake equal to or greater than two and/or heart/CL ≥1.5 in the first hour or ≥ 1.3 in the third hour.^7 – 11^

### Speckle tracking echocardiography protocol

Patients underwent echocardiographic examination using a Philips CX50 portable machine, version 5.0.2 (IE 33 system, Philips Medical System), with a S4-2 sector probe. The images were acquired by an investigator experienced in the method of strain assessment, who was blinded for the PYP scintigraphy results. The Automated Cardiac Motion Quantification (aCMQ) and the QLAB tool of the CX50 were used to calculate left ventricular global longitudinal strain. Image acquisition and interpretation were carried out according to the American Society of Echocardiography recommendations.^12^Global longitudinal strain (GLS) measurements and analysis for an image known as apical sparing were performed in the two-, three-, and four-chamber views. GLS values ≥-18% were considered normal.^12 , 13^

### Subclinical cardiac involvement

Subclinical involvement was defined as visual score (semiquantitative) ≥2 and/or a H/CL ratio ≥ 1.5 at one hour or ≥ 1.3 three hours after radiotracer infusion (quantitative score) and/or GLS ≤ −17%by transthoracic echocardiogram.

### Statistical analysis

Data were collected and tabulated. The Statistical Package for the Social Sciences (SPSS), version 25.0 (IBM) was used for the analyses. Categorical variables were described as percentage (proportion), and nonparametric continuous variables as median and interquartile. Categorical variables were compared by Fisher's exact test and nonparametric continuous variables by Mann-Whitney test. The Shapiro-Wilk test was used to assess the normality of data distribution. A ROC curve was used to compare the diagnostic accuracy between a numerical and a categorical variable. A p<0.05 was considered statistically significant in all analyses.

## Results

The sample was composed of 23 patients with no cardiovascular symptoms ( Table 1 ).

**Table 1:** Demographic and echocardiographic characteristics of participants

Characteristics		Sample size (n=23)
**Age (years)**		51 (IQR * 37-57)
**Sex, n (%)** **Race, n (%)**		Female. 15 (65)
		Pardo. 12 (52.2)
		Black. 7 (30.4)
		White. 4(17.4)
**Type of mutation, n° (%)**		Val142IIe. 9 (43)
		Val50met. 8 (35)
		IIe127Val. 5 (22)
**Asymptomatic carriers, n° (%)**		**14 (61)**
	FAP, n (%)	9 (39.1)
	SAH, n (%)	9 (39.1)
	DM, n (%)	4 (17.4)
	DLP, n (%)	6 (26)
	Current smoker, n° (%)	2 (8.7)
	Use of tafamidis, n° (%)	8 (34.8)
**EF (%)**		**69 (IQ 66 - 72)**
	LVEED (mm)	44 (IQ 40 - 48)
	LVESD (mm)	27(IQ 26 - 30)
	Interventricular septum (mm)	9 (IQ 8 -10)
	Posterior wall (mm)	8 (IQ 8 - 9)
**LVMI (g/m^2^)**		**72 (IQ 62 - 81)**
	Left atrial volume (ml/m^2^)	29 (IQ 26 - 31)
	Mitral E-wave velocity (cm/s)	80 (IQ 61 – 92)
	Mitral A-wave velocity (cm/s)	68 (IQ 59 – 80)
	E/A ratio	1.0 (IQ 0.9 – 1.6)
	Tissue Doppler septal e’ velocity (cm/s)	9.0 (IQ 8 – 10)
	Tissue Doppler lateral e’ velocity (cm/s)	11 (IQ 10 – 12)
	Mean E/e’	8.1 (IQ 6.8 – 9.0)
**Global longitudinal strain (%)**		- 19 (IQ – 16% a – 20%)

* FAP: familial amyloid polyneuropathy; SAH: systemic arterial hypertension; DM: diabetes mellitus; DLP: dyslipidemia; EF: ejection fraction; LVEED: left ventricular end-diastolic diameter: LVESD; left ventricular end-systolic diameter; LVMI: left ventricular mass index; *IQR: interquartile range.

Based on PYP scintigraphy and strain echocardiography results, 14 patients (60.9%) did not show abnormal cardiovascular findings. Nine (39.1%; 95%CI 29% - 49%) met the criteria for cardiac involvement, six (26%) using the strain criterion alone, one (4.3%) using the PYP criterion alone and two (8.7%) showed cardiac alterations based on both PYP and strain criteria ( Figure 2 ).

**Figure 2:** Prevalence of subclinical cardiac involvement by diagnostic method: pyrophosphate scintigraphy and strain echocardiography.

Nineteen (82.6%) showed indeterminate results by PYP scintigraphy (degree of uptake 1), 18 had a H/CL ratio ≤ 1.4 in the first hour after PYP administration (non-diagnostic). The presence of blood pool was detected in three patients (SPECT carried out in the first and in the third hour after PYP injection).

There was no association between having FAP and being an asymptomatic carrier in the assessment of the presence of cardiac involvement by strain echocardiography or PYP scintigraphy (p=0.19). Among the 14 asymptomatic carriers, four (17.45) had evidence of preclinical cardiac involvement, and among the patients with FAP (n=9), five (21.7%) had suggestive subclinical compromise ( Figure 3 ).

**Figure 3:** Bar graph representing the presence of cardiac involvement in patients with familial amyloid polyneuropathy; Fisher's exact test.

There was no difference in the prevalence of hypertension, diabetes mellitus (DM), dyslipidemia (DLP), and smoking habit between patients with and without reduced strain ( Table 2 ).

**Table 2:** Characteristics of participants by global longitudinal strain (GLS) values

Caracteristics		GLS ≥ −18%	GLS ≤ −17%	p Value
SAH, n° (%)		7 (43.8)	2 (25.0)	0.41
DM, n° (%)		4 (25.0)	0	0.27
DLP, n° (%)		6 (37.5)	0	0.12
Current smoker, n (%)		2 (12.5)	0	0.5
* **Diastolic function parameters**				
	Left atrial volume (mL/m^2^)	29 (26-31)	29 (28-31)	0.62
	E velocity (cm/s)	84.5 (64.2-97.2)	68.0 (61-86)	0.37
	A velocity (cm/s)	67.5 (59-79.5)	69.0 (59-92)	0.82
	E/A ratio	1.1 (0.96-1.77)	1.0 (0.87-1.19)	0.37
	Septal e’ velocity (cm/s)	9.0 (8.3-11)	8.0 (8-8)	**0.027**
	Lateral e’ velocity(cm/s)	11 (10-13.5)	10 (10-11)	0.10
	Mean E/e’	8.1 (6.8-9.0)	7.4(6.8-9.7)	0.99

* Diastolic function variables were described as median and interquartile range; differences between the groups were assessed by Fisher's exact test for categorical variables and by Mann-Whitney test for continuous variables; SAH: systemic arterial hypertension; DM: diabetes mellitus; DLP: dyslipidemia.

The determinant variables of diastolic function (Doppler mitral E-wave velocity and A-wave velocity, E/A ratio, septal and lateral e’ velocity by tissue Doppler and mean E/e’) were compared between patients with and without reduced strain ( Table 2 ).

Tissue Doppler septal e’ velocity was the only variable that showed statistically significant difference between the groups, with an area under the ROC curve of 0.80 (95%CI 0.61 – 0.98; p = 0.027). An e’ velocity equal to or lower than 8.5 cm/s showed the best diagnostic accuracy in predicting reduced strain ( Figure 4 ).

**Figure 4:** ROC curve representing the diagnostic accuracy between septal e’ velocity and myocardial strain; *AUC: area under the curve; ROC: Receiver Operating Characteristic.

There was no significant association between the type of mutation and the presence of preclinical cardiac involvement ( Figure 5 ). Also, there was no association between the use of tafamidis and a lower cardiac involvement degree (37.5% in patients who used tafamidis versus 40% in patients who did not use tafamidis, p = 0.90) ( Figure 6 ).

**Figure 5:** Distribution of mutations in patients with and without cardiac involvement.

**Figure 6:** Frequency of cardiac involvement in patients using and not using Tafamidis.

## Discussion

Subclinical cardiac involvement, defined as a GLS reduction ≤ −17% and/or a visual score ≥ 2 or a H/CL ≥ 1.5 in one hour or ≥ 1.3 three hours after PYP injection, was observed in 39% (n=9) of the 23 participants of the study. Six patients showed altered GLS values alone, two met the scintigraphic and echocardiographic criteria, and one using the PYP criterion alone ( Central Illustration ). Blood pool, which is known the cause false-positive results,^7 – 9^was found in three patients after SPECT.

We found no significant association (p=0.19) in the analysis of the presence of cardiac involvement in patients with FAP (n=5) and in asymptomatic patients (n=4). In the present study, three types of mutation were identified, one of them (Val142lle) more commonly related to cardiac clinical manifestations.^3 , 14 – 16^However, in the analysis of the categorical variable cardiac involvement, there was no significant difference between the mutations. Most patients in our sample were women (65%), which must be highlighted since the TTR variant (or hereditary) has been predominantly found among men.^3 , 17 -, 20^Septal e’-wave velocity, assessed by tissue Doppler echocardiography, was the echocardiographic diastolic function parameter found to be positively associated with altered myocardial strain. A value ≤ 8.5 cm/s achieved the best accuracy, with an area under the ROC curve of 0.80 (95%CI 0.61-0.98) and a p-value of 0.027. Although this is a still exploratory result, it may indicate that septal e’ velocity values ≤ 8,5cm/s are associated with a presymptomatic cardiac involvement and may be helpful for patient screening when strain measurement is not available. Since septal e’ velocity is an easy, fast, routine echocardiographic measure, we believe this information has potential practical applicability.

In our sample, there was no difference in the prevalence of hypertension, DM, DLP, or smoking between the patients with and without reduced strain. There was no association between the type of mutation and the presence of preclinical cardiac involvement. Also, there was no association between the use of tafamidis (20mg/day) and a lower prevalence of cardiac involvement (37.5% versus 40.0%, p = 0.90). It is worth remembering that the dose of 20mg/day is well indicated in the neurological form of TTR amyloidosis. In addition, the treatment period with tafamidis was up to six months, which may not have been sufficient to demonstrate any cardiovascular benefit.

Among the preestablished criteria to identify subclinical myocardial involvement, GLS was the most altered parameter; however, apical sparing, which is the typical image of CA, was not found in any of the subjects.

Echocardiogram with strain is known to be sensitive method for the diagnosis of preclinical cardiac involvement.^13^Although the technique has been consolidated as an analysis tool of systolic function, notably in cardio-oncology,^14^data on its role in ATTR with cardiomyopathy are less robust.^2 , 3^Studies have tried to demonstrate the importance of an early diagnosis in CA using longitudinal and circumferential strain, scintigraphy with bone markers, and myocardial T1 mapping and extracellular volume quantification by cardiac magnetic resonance imaging.^1 , 2^

The natural history of asymptomatic carriers of the TTR mutation and of patients with the neurological disease alone has been fully elucidated.^1^Types of mutation, degree of penetrance and environmental factors actively interact in this process.^1 , 2^What noninvasive method is the most sensitive to detect subclinical involvement is debatable and still uncertain, and consequently, it is a subject of intense research and great interest. Biomarkers like troponin and BNP, resting electrocardiogram and conventional electrocardiogram are little accurate instruments in this population.^1 , 2 , 15^A recently published study used measurements of retinol-binding protein 4 and misfolded TTR in attempt to detect subclinical TTR CA.^1^Quarta et al.^16^showed that some echocardiographic changes, like increased left ventricular posterior wall, E/e’ and abnormal GLS, are more prevalent in Val142Ile variant carriers.^16^In 2020, Sinha et al.^17^showed that Val142Ie had lower systolic circumferential strain as compared with controls. However, important limitations of these studies include the fact that no other specific techniques were conducted for the diagnosis of this subclinical condition, such as biopsy, cardiac resonance, or PYP scintigraphy.^1 , 16 , 17^In 2017, Haq et al.^18^investigated whether PYP scintigraphy could detect early amyloid deposit in the heart of 40 patients with mutant TTR genopositivity but without HF. In addition to measurements of troponin I and BNP, all patients underwent transthoracic echocardiogram. Of the TTR mutation carriers (n=12), 10 (84%) showed some degree of myocardial uptake, and seven (58%) revealed grade 2 or 3 of PYP uptake. The H/CL ratio was 1.5 ± 0.4 in asymptomatic carriers and 1.2 ± 0.1 in those with nonamyloid HF and preserved ejection fraction, with a p-value of 0.02. The authors concluded that abnormal PYP uptake may be the first detectable manifestation in asymptomatic carriers of TTR mutations, preceding changes in biomarkers levels, electrocardiogram and echocardiogram. In this study, cardiac magnetic resonance and echocardiogram with strain were not analyzed.^18^

In our study, both PYP and strain were analyzed concomitantly, which is a more contemporary approach as compared with previous publications. Due to its practicality, safety and no use of radiation, the proposal to add the echocardiogram in the diagnostic investigation of asymptomatic individuals seems promising. Although PYP was positive in three participants, a reduction in GLS was more common. These data add new information about the usefulness and importance of myocardial strain in the management of these patients and suggest the study of myocardial strain as one of, maybe the most sensitive, tool in the investigation of individuals without overt cardiovascular symptoms.

In addition, it is worth pointing out that in three asymptomatic patients, although the electrocardiogram and echocardiogram results were normal, PYP scintigraphy findings were altered, indicating the presence of amyloid material in the heart. Based on the clinical practice, asymptomatic carriers of TTR mutations with FAP are not routinely investigated for the presence of myocardial amyloid deposition, which possibly should be revised.

In addition, in FAP patients, a concomitant subclinical cardiac involvement was not observed. This may be explained either because of absence of amyloid material or because they are actually two different diseases, which contrasts with previous literature data.^18^As investigations proceed, more people are diagnosed, and more relatives of the index cases have been seen in the offices, with their agonies and questions.^1 – 4 , 19^Therefore, the best follow-up strategy of these patients, including the most sensitive screening tests, needs to be found and, in this regard, we believe this is a groundbreaking study.

Carriers of TTR mutations are at risk to develop HF at any time of life.^1 – 4^Proactive behaviors, supported by high-quality science, can change the natural history of this progressive and often inevitable disease,^1 – 4 , 19^and this is exactly our proposal and our man point.

Here we present a study with the perspective of helping discriminating individuals without a subclinical myocardial involvement from those with some degree of myocardial amyloid deposit. A more regular follow-up, focused on the development of signs and symptoms of this condition should be implemented. However, we still do not know the most sensitive noninvasive method to diagnose this subclinical cardiac involvement and, based on the results, the best strategy to follow.^1 – 4^Despite considerable advances in the diagnosis of ATTR cardiomyopathy,^7 – 11^the most effective management of asymptomatic TTR mutation carriers is still unclear.^1 – 4^Larger studies are needed to consolidate GLS and PYP scintigraphy as protagonists in the diagnosis of asymptomatic carriers and of neurological patients.

Similarly to other echocardiographic techniques, strain echocardiography requires an adequate acoustic window in addition to being examiner- and device-dependent.^12 , 13^In addition, a limitation of the method, and consequently of this study, is its intra- and interobserver variability,^12 , 13^which reduces both accuracy and reproducibility of this technique. Also, the technique was conducted by the same echocardiographist, which, despite his experience, may be considered another limitation of the study. Due to our sample of 23 patients, caution is needed in extrapolating our results. Results by chance are more common in small samples and the risk of hasty conclusions is intrinsic to type I error. Also, it is worth mentioning that, despite the sample size limitation, TTR amyloidosis is a rare or uncommon disease and hence with inherent sampling limitations.

## Conclusion

Subclinical cardiac involvement was frequently observed in a sample of patients with TTR mutations, without cardiovascular symptoms or changes in cardiological screening tests. Reduced left ventricular GLS was the most common parameter in PYP scintigraphy. There was no relationship between the presence of amyloidotic polyneuropathy and subclinical involvement. The type of mutation was not associated with early cardiac involvement and, in our study sample, the use of tafamidis was not associated with a lower prevalence of subclinical cardiac involvement.

Larger studies are needed to consolidate the global strain and PYP scintigraphy as protagonists in the diagnostic investigation of asymptomatic carriers of TTR mutations and those with the neurological form of the disease.
